# Dr. W. D. Southwick

**Published:** 1896-10

**Authors:** 


### NECROLOGY.

DR. W. D. SOUTHWICK, graduate of the American Veterinary College of New York City, Class of 1887, died at Woonsocket, R. I., on July 13, 1896. His illness (typhoid fever) lasted over a period of five weeks. Dr. Southwick was one of the best-known veterinary surgeons in Rhode Island. He was a graduate of Blackstone High School, Class of 1883. After his graduation in 1887 he entered upon the pursuit of his profession at Woonsocket, and immediately received substantial support and recognition from the people of his community. He was an indefatigable worker, and exhibited a keen interest in bovine tuberculosis and the use of tuberculin as a diagnostic agent. His intelligent use of measures necessary to stamp out diseases among cattle won for him a high place among the stockowners of his own and adjacent States. His death has brought forth a widespread expression of regret.

